# Supplementary material for: Preservation of latest Cretaceous (Maastrichtian)—Paleocene frogs (Eorubeta nevadensis) of the Sheep Pass Formation of east-central Nevada and implications for paleogeography of the Nevadaplano
Source: PeerJ. 2020 Jul 3;8:e9455. doi: 10.7717/peerj.9455 (PMC7341540; doi:10.7717/peerj.9455)
Supplement: Data S1 [file peerj-08-9455-s001.docx]

Raw Data: Specimens referred to in text with repository

Sierra College Natural History Museum

SCNHM VAF 3- Nearly complete frog from Taphonomic Mode 1, Member B of Sheep Pass Formation

SCNHM VAF 4- Nearly complete frog from Taphonomic Mode 4, Member C of Sheep Pass Formation

SCNHM VAF 11- A piece of the frog bonebed of Taphonomic Mode 5, Member C of Sheep Pass Formation

SCNHM VAF 26 A- Nearly complete frog with associated ostradods from Taphonomic Mode 2, Member B of Sheep Pass Formation

Carnegie Museum of Natural History

CM 89263- Nearly complete frog from Taphonomic Mode 4, Member C of Sheep Pass Formation
